# ICONIC study—conservative versus conventional oxygenation targets in intensive care patients: study protocol for a randomized clinical trial

**DOI:** 10.1186/s13063-022-06065-7

**Published:** 2022-02-13

**Authors:** C. C. A. Grim, L. I. van der Wal, H. J. F. Helmerhorst, D. J. van Westerloo, P. Pelosi, M. J. Schultz, E. de Jonge, for the ICONIC Investigators and PROVE Network, M. R. del Prado, J. Wigbers, M. J. Sigtermans, L. Dawson, P. L. J. van der Heijden, E. Y. Schriel-van den Berg, B. G. Loef, A. C. Reidinga, E. de Vreede, J. Qualm, E. C. Boerma, H. Rijnhart-de Jong, M. Koopmans, A. D. Cornet, T. Krol, M. Rinket, J. W. Vermeijden, A. Beishuizen, F. J. Schoonderbeek, J. van Holten, A. M. Tsonas, M. Botta, T. Winters, J. Horn, F. Paulus, M. Loconte, D. Battaglini, L. Ball, I. Brunetti

**Affiliations:** 1 Department of Intensive Care, Leiden University Medical Center, Albinusdreef 2, 2333 ZA Leiden, The Netherlands; 2 Department of Anesthesiology, Leiden University Medical Center, Albinusdreef 2, 2333 ZA Leiden, The Netherlands; 3 Department of Surgical Sciences and Integrated Diagnostics, San Martino Policlinico Hospital, IRCCS for Oncology and Neurosciences, Largo R. Benzi 10, 16132 Genoa, Italy; 4 Department of Anesthesia and Intensive Care, San Martino Policlinico Hospital, IRCCS for Oncology and Neurosciences, Largo R. Benzi 10, 16132 Genoa, Italy; 5 Department of Intensive Care, Amsterdam University Medical Centre, Location AMC, Amsterdam, The Netherlands; 6 Mahidol – Oxford Tropical Medicine Research Unit (MORU), Mahidol University, Bangkok, Thailand; 7 Nuffield Department of medicine, University of Oxford, Oxford, UK

**Keywords:** Mechanical ventilation, Oxygen inhalation therapy, Intensive care, Oxygen, Clinical trial

## Abstract

**Background:**

Oxygen therapy is a widely used intervention in acutely ill patients in the intensive care unit (ICU). It is established that not only hypoxia, but also prolonged hyperoxia is associated with poor patient-centered outcomes. Nevertheless, a fundamental knowledge gap remains regarding optimal oxygenation for critically ill patients. In this randomized clinical trial, we aim to compare ventilation that uses conservative oxygenation targets with ventilation that uses conventional oxygen targets with respect to mortality in ICU patients.

**Methods:**

The “Conservat*I*ve versus *CON*ventional oxygenation targets in *I*ntensive *C*are patients” trial (ICONIC) is an investigator-initiated, international, multicenter, randomized clinical two-arm trial in ventilated adult ICU patients. The ICONIC trial will run in multiple ICUs in The Netherlands and Italy to enroll 1512 ventilated patients. ICU patients with an expected mechanical ventilation time of more than 24 h are randomized to a ventilation strategy that uses conservative (PaO_2_ 55–80 mmHg (7.3–10.7 kPa)) or conventional (PaO_2_ 110–150 mmHg (14.7–20 kPa)) oxygenation targets. The primary endpoint is 28-day mortality. Secondary endpoints are ventilator-free days at day 28, ICU mortality, in-hospital mortality, 90-day mortality, ICU- and hospital length of stay, ischemic events, quality of life, and patient opinion of research and consent in the emergency setting.

**Discussion:**

The ICONIC trial is expected to provide evidence on the effects of conservative versus conventional oxygenation targets in the ICU population. This study may guide targeted oxygen therapy in the future.

**Trial registration:**

Trialregister.nl NTR7376. Registered on 20 July, 2018.

## Administrative information

**Table:** 

Title {1}	ICONIC-Conservative versus conventional oxygenation target in Intensive Care patients: protocol for a randomized clinical trial
**Trial registration {2a and 2b}.**	Trial number: NTR7376 on trialregister.nl
**Protocol version {3}**	Version 11 13 February 2020
**Funding {4}**	This work is part of the research program Replication Studies with project number 401.16.009, which is (partly) financed by the Dutch Research Council (NWO).
**Author details {5a}**	C.C.A. Grim* ^1,2^, L.I. van der Wal* ^1,2^, H.J. F. Helmerhorst ^1,2^, D.J. van Westerloo ^1^, P. Pelosi ^3,4^, M.J. Schultz ^5,6,7^, E. de Jonge ^1^, for the ICONIC Investigators and PROVE Network *contributed equally 1. Department of Intensive Care, Leiden University Medical Center, Albinusdreef 2, 2333 ZA Leiden, The Netherlands 2. Department of anesthesiology, Leiden University Medical Center, Albinusdreef 2, 2333 ZA Leiden, The Netherlands 3. Department of Surgical Sciences and Integrated Diagnostics, San Martino Policlinico Hospital, IRCCS for Oncology and Neurosciences, Largo R. Benzi 10, 16132, Genoa, Italy 4. Department of Anesthesia and Intensive Care, San Martino Policlinico Hospital, IRCCS for Oncology and Neurosciences, Largo R. Benzi 10, 16132, Genoa, Italy 5. Department of Intensive Care, Amsterdam University Medical Centre, Location AMC, Amsterdam, The Netherlands 6. Mahidol – Oxford Tropical Medicine Research Unit (MORU), Mahidol University, Bangkok, Thailand 7. Nuffield Department of medicine, University of Oxford, Oxford, United Kingdom
**Name and contact information for the trial sponsor {5b**	E. de Jonge Leiden University Medical Center Department of Intensive Care Albinusdreef 2, 2333 ZA Leiden, The Netherlands Telephone: + 31 71 5265018 E-mail: e.de_jonge@lumc.nl
**Role of sponsor {5c}**	The sponsor co-designed the ICONIC trial, leads the collection, management, analysis and interpretation of data. The sponsor has a leading role in writing the report and shall be responsible for submitting the ICONIC trial for publication. The funder will have no authority over collection, management, analysis and interpretation of data, writing of the report, and the decision to submit for publication.

## Introduction

### Background and rationale {6a}

Arterial oxygenation may be influenced by different factors, including lung function, lung mechanics, ventilator settings, hemodynamics, and the amount of oxygen administered. The risks of hypoxia are well-established, and prolonged exposure to severe hyperoxia has also been shown to induce lung injury [1–4]. In two meta-analyses, arterial hyperoxia and liberal use of oxygen therapy were associated with hospital mortality and poor functional outcome in various subsets of critically ill patients [5, 6]. However, the retrospective nature of the meta-analyzed studies hamper general acceptance of lower target ranges and supraphysiological oxygenation is still frequently pursued in order to avoid hypoxemia. In a Dutch study, the nadir for unadjusted mortality was retrospectively determined at oxygenation levels of 110–150 mmHg [7], but pilot data suggest that more conservative oxygenation targets may also be safe and even improve clinical outcomes [8].

Accordingly, a fundamental knowledge gap regarding optimal oxygenation has been recognized in international literature [9–15].

In a randomized clinical trial on optimal oxygenation in ICU patients that was published in 2016, improved survival was demonstrated in patients who received oxygen according to the conservative strategy (PaO_2_ targeting 70–100 mmHg or arterial oxyhemoglobin saturation (SpO_2_) targeting 94–98%) in comparison to a conventional control group (PaO_2_ up to 150 mmHg or SpO_2_ targeting 97–100%) [16]. This trial was the first randomized clinical study to demonstrate a potential harm of liberal oxygen administration, which earlier had been suggested by observational and preclinical studies [17–21].

However, after this first RCT, three comparable trials have been completed that did not support the previous findings that favored lower oxygenation targets [22–24]. Thus, uncertainty still exists on optimal oxygenation targets in ICU patients.

### Objectives {7}

As a replication study, we have set up a multicenter trial comparing conservative and conventional oxygenation targets in ICU patients, to confirm findings from a previous study that showed improved survival in ICU patients treated with lower oxygenation targets [16]. To that end, we applied similar in- and exclusion criteria and similar oxygenation targets.

### Trial design {8}

The ICONIC study is an investigator-initiated, multicenter, international, open-label, parallel, 1:1 randomized clinical two-arm equivalence trial in mechanically ventilated ICU patients.

## Methods: participants, interventions, and outcomes

### Study setting {9}

Patients are recruited from ICUs from participating hospitals, academic and non-academic, in Europe. The participating hospitals are as follows:

- Leiden University Medical Centre, Leiden, The Netherlands
- Medisch Centrum Leeuwarden, Leeuwarden, The Netherlands
- Martini Hospital, Groningen, The Netherlands
- Amsterdam University Medical Centre, Amsterdam, The Netherlands
- Ikazia Hospital, Rotterdam, The Netherlands
- Reinier de Graaf Gasthuis, Delft, The Netherlands
- Medisch spectrum Twente, Enschede, The Netherlands
- Diakonessenhuis, Utrecht, The Netherlands
- San Martino Hospital, Genoa, Italy

### Eligibility criteria {10}

#### Inclusion criteria

In order to be eligible to participate in this study, a subject must meet all of the following criteria:

- Age ≥ 18 years
- Admission to an ICU participating in this study
- Need for intubation and mechanical ventilation
- Expected mechanical ventilation time of 24 h or longer
- Inclusion within 2 h after start of invasive ventilation in the ICU or if previously intubated and ventilated within 2 h after admission to the ICU

#### Exclusion criteria

A potential subject who meets any of the following criteria will be excluded from participation in this study:

- Readmission to the ICU within the same hospital admission
- Prior ICONIC study inclusion
- Invasive ventilation longer than 12 h directly preceding admission
- Decision to withhold life-sustaining treatment at the time of inclusion
- Acute respiratory distress syndrome (ARDS) with a PaO_2_/FiO_2_ ratio less than 150 mmHg
- Acute decompensation of chronic obstructive pulmonary disease (COPD) and chronic hypoxemia
- Use of home oxygen therapy
- Severe not rapidly reversible low cardiac output shock (for example: cardiac index ≤ 2 L/min/m^2^)
- Documented severe pulmonary hypertension
- Veno-arterial extracorporeal membrane oxygenation (VA-ECMO)
- Underlying disease indication for hyperoxygenation (for example: carbon monoxide intoxication, decompression sickness, gas embolism)
- Severe anemia (hemoglobin < 4.0 mmol/l) that is not rapidly reversible (e.g., if blood transfusions are not possible or not allowed for religious reasons)
- Uncontrollable intracranial hypertension
- Participation in other interventional trials which could influence ICONIC study intervention and/or endpoints
- Suspected or confirmed pregnancy

### Who will take informed consent? {26a}

Informed consent will be obtained according to local legal regulations. Informed consent will be obtained, if possible, prior to start of intervention. However, due to the emergency setting of this trial, this will occur in the minority of subjects. For the majority of subjects, inclusion will take place in an emergency setting when the patient is incapacitated and deferred consent from a proxy will be obtained as soon as possible. Information about the trial will be given by the treating physician to the proxy. After deferred proxy consent is obtained, decisional capacity of the participant will be assessed frequently and when regained during the ICU stay deferred subject consent must be obtained.

If the patient dies before informed consent or deferred (proxy or subject) consent is obtained, the study data will be used. The Dutch central committee of research in humans (Centrale Commissie Mensgeboden Onderzoek (CCMO)) states that legal representation of a patient ends after death and that therefore the obligation to obtain signed consent no longer applies after death of the patient [25].

### Additional consent provisions for collection and use of participant data and biological specimens {26b}

This trial does not involve collecting biological specimens for storage.

## Interventions

### Explanation for the choice of comparators {6b}

The comparators were chosen based upon previously found oxygenation targets associated with greater survival in ICU patients [8, 26] and to have sufficient contrast in PaO_2_ between the two randomization groups.

### Intervention description {11a}

In patients randomized to the “conservative-targets” arm, oxygenation will be targeted at PaO_2_ 55–80 mmHg (7.3–10.7 kPa). Because PaO_2_ is not continuously measured, oxygenation targets can be steered on SpO_2_ in between PaO_2_ measurements. Corresponding SpO_2_ to conservative PaO_2_ targets needs to be determined per individual patient (usually approximately 91–94%).

Patients randomized to the “conventional-targets” arm, oxygenation will be targeted at PaO_2_ between 110 and 150 mmHg (14.7–20 kPa). Corresponding SpO_2_ to conventional PaO_2_ targets will also be determined per individual patient (usually approximately 96–100%).

#### Invasive ventilation

The allowed ventilation modes are volume-controlled ventilation, pressure-controlled ventilation, pressure support ventilation, closed loop ventilation, and combined modes. Furthermore, INTELLiVENT-ASV (Hamilton Medical AG, Bonaduz, Switzerland) is allowed with the automatic oxygenation (FiO_2_ and PEEP) adjustment turned off.

The inspired oxygen fraction (FiO_2_) and positive end-expiratory pressure (PEEP) values are determined and titrated by means of the pre-specified and randomly assigned oxygenation targets. The respiratory rate is adjusted to maintain a blood pH of 7.20 to 7.45. In case of metabolic acidosis or alkalosis, a lower or higher than normal PaCO_2_ can be accepted, left to the discretion of the attending physician. The lowest level of PEEP is 5 cmH_2_O; recommended FiO_2_–PEEP combinations are provided in Table 1. Deviation from the table is allowed in individual patients when indicated and is left to the discretion of the attending physician. Recruitment maneuvers are allowed, when deemed necessary by the attending physician.

**Table 1:** Recommended combinations of FiO_2_ and PEEP. Deviation from the table is allowed in individual patients when indicated and is left to the discretion of the attending physician

FiO_**2**_	PEEP (cmH_**2**_O)
0.21	5
0.30	5
0.40	5
0.40	8
0.50	8
0.50	10
0.60	10
0.70	10
0.70	12
0.70	14
0.80	14
0.90	16
0.90	18
1.00	18
1.00	20
1.00	22
1.00	24

In both arms, tidal volume is titrated per predicted bodyweight (PBW), which is calculated according to a previously used formula: 50 + 0.91 × (centimeters of height − 152.4) for males and 45.5 + 0.91 × (centimeters of height − 152.4) for females. Tidal volumes are targeted at 6–8 ml/kg PBW.

#### Weaning

Daily assessment of the ability to breathe with pressure support ventilation is required as soon as FiO_2_ ≤ 0.4 or when the PEEP level and FiO_2_ level are lower than the day before.

In addition, the ventilator can be switched to pressure support ventilation at any moment if the attending nurse or physician considers the patient awake enough to breathe with pressure support ventilation. Assessment of the ability to breathe with pressure support is also required in case patient–ventilator asynchrony is noticed (ineffective breathing; double triggering, use of accessory respiratory muscles). A patient is assumed to be ready for extubation when the following criteria are met for at least 30 min, and the final decision for extubation is made by the attending physician:

- Responsive and cooperative
- Adequate cough reflex
- PaO_2_/FiO_2_ of > 200 mmHg with FiO_2_ ≤ 40%
- Respiratory rate of 8 to 30 per minute
- No signs of respiratory distress (i.e., marked accessory muscle use, abdominal paradox, diaphoresis, marked dyspnea)
- Pressure support level < 8 cmH_2_O
- Hemodynamically stable (systolic blood pressure 80 to 160 mmHg and heart rate 40 to 130/min) and no uncontrolled arrhythmia
- Temperature > 36.0 and < 38.5 °C

If a patient is able to breathe without assistance but subsequently requires additional ventilation within 28 days after randomization, the same oxygenation target protocol is resumed.

#### After invasive ventilation

When a patient is extubated, the PaO_2_ targets should still be pursued within the type of oxygen support for which the patient has a medical indication. High-flow nasal oxygen or non-invasive ventilation should not be started solely for the ICONIC study PaO_2_ targets, because this could influence duration of ICU admission. If this means the PaO_2_ targets are not achieved after extubation, this should be accepted. The following rules apply:

- For patients randomized to the conventional oxygenation target: always give a nasal cannula with 5 L of oxygen, except if PaO_2_ > 150 mmHg (> 20 kPa).
- For patients randomized to the conservative target: preferably no oxygen therapy, except if PaO_2_ < 55 mmHg (< 7.3 kPa).

### Criteria for discontinuing or modifying allocated interventions {11b}

Subjects can leave the study at any time for any reason if they wish to do so without any consequences. The investigator can decide to withdraw a subject from the study for urgent medical reasons. When deferred consent is not obtained after randomization and provisional inclusion of a patient or when a patient withdraws consent. The replacement of the randomization subject will be done in the automated randomization scheme.

**Fig. 1:**
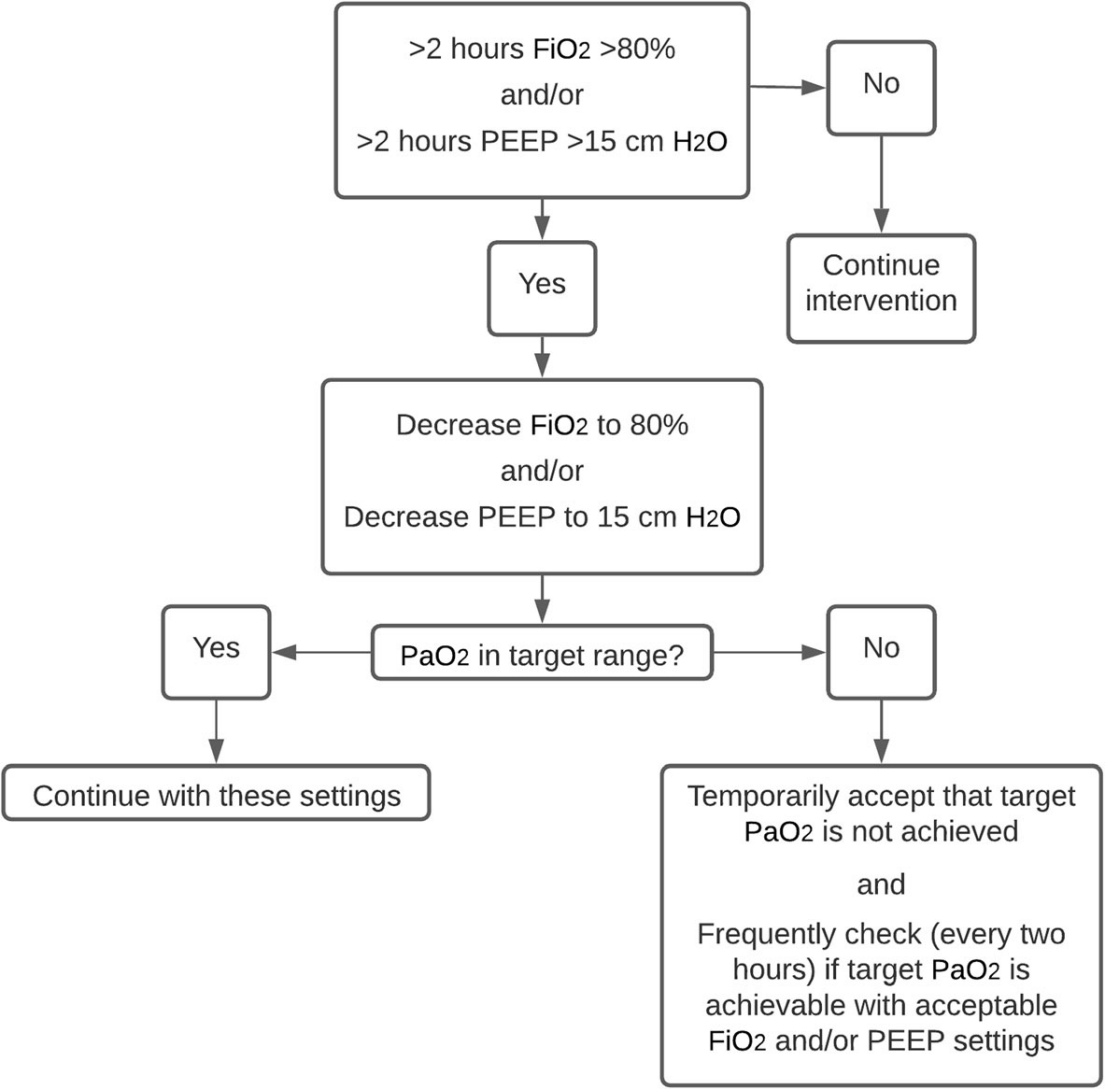
Flowchart high FiO_2_ and/or high PEEP

To avoid prolonged exposure to very high inspiratory oxygen concentrations, the allocated intervention can temporarily be modified in the conventional PaO_2_ target group when FiO_2_ is above 80% for more than 2 h and/or PEEP is above 15 cmH_2_O for more than 2 h. In order to provide guidance when clinicians are in a situation with high inspiratory oxygen concentrations, we created a flowchart (Fig. 1).

### Strategies to improve adherence to interventions {11c}

At least one blood gas analysis per shift (three per 24 h) will be required while mechanically ventilated.

If a participating ICU has difficulty adhering to the oxygenation targets and there is risk of overlap between the groups, the “aiming point PaO_2_” provides guidance to the bedside clinicians:

- Conservative arm aiming point PaO_2_ 60 mmHg (8 kPa)
- Conventional arm aiming point PaO_2_ 135 mmHg (18 kPa)

### Relevant concomitant care permitted or prohibited during the trial {11d}

Among other concomitant care, sedation, selective oropharyngeal or digestive tract decontamination, thrombosis prophylaxis, fluid regimens, and nutrition follow the local guidelines in each participating ICU and are permitted during the trial.

### Provisions for post-trial care {30}

No provisions or restrictions are applicable for post-trial care. The sponsor has an insurance which is in accordance with the legal requirements in the Netherlands (Article 7 WMO). This insurance provides cover for damage to research subjects through injury or death caused by the study. The insurance applies to the damage that becomes apparent during the study or within 4 years after the end of the study.

### Outcomes {12}

The primary endpoint is all-cause mortality at 28 days after randomization. The secondary study endpoints are as follows:

- The number of ventilator-free days and alive at day 28, defined as the number of calendar days from day 1 to day 28, the patient is alive and breathes without assistance of the mechanical ventilator. Ventilator-free days are according to the definitions by the Dutch National Intensive Care Evaluation (NICE) registry [27].
- ICU length of stay (LOS)
- Hospital LOS
- ICU mortality
- Hospital mortality
- 90-day mortality
- Ischemic events (cardiac, neurological and peripheral)

Follow-up (in participating subjects from the Netherlands):

- Quality of life at 6 and 12 months
- Patient opinion of research and consent in the emergency setting at 6 months after randomization

### Participant timeline {13}

**Fig. 2:**
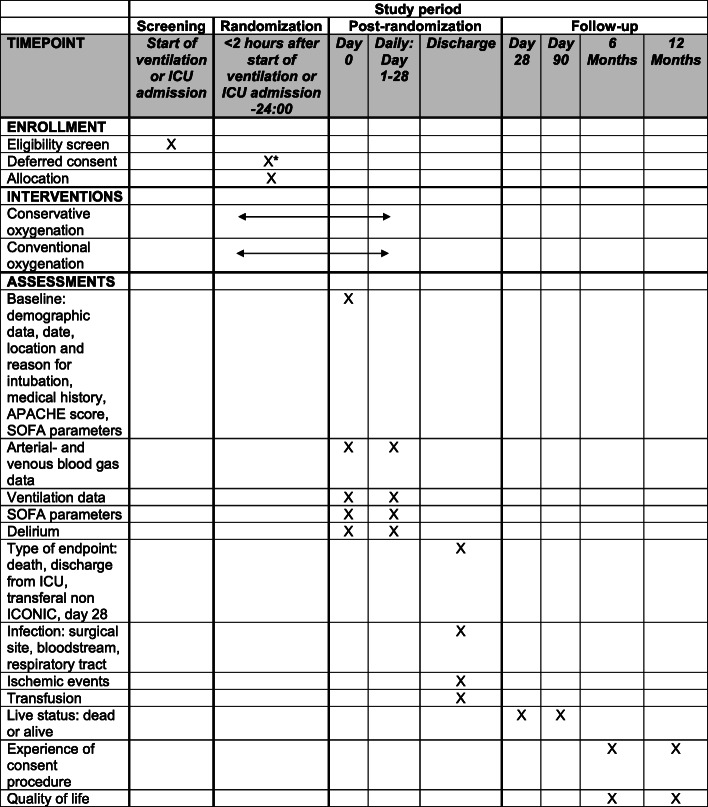
Schedule of enrollment, intervention, and assessments

Participant timeline is shown in Fig. 2.

### Sample size {14}

Based on an expected mortality in the control group of 24% (source: Dutch NICE foundation; NICE online [27]), we will include 1512 patients to detect an absolute difference in mortality of 6% (2-sided, alpha 0.05, power 80%, similar allocation of subjects to each group and corrected for 4% dropouts). The choice of 6% was motivated by the difference of 8% found in a previous trial [16] comparing conventional to conservative oxygenation targets and what could be considered clinically acceptable.

### Recruitment {15}

All patients admitted to participating ICUs or intubated on participating ICUs will be screened for eligibility.

## Assignment of interventions: allocation

### Sequence generation {16a}

Randomization sequence is generated by a dedicated computer randomization software program (Castor EDC, Amsterdam, The Netherlands) using variable block sizes and is stratified per participating center.

Details of blocking are provided in a separate document that is unavailable to those who enroll participants or assign interventions.

### Concealment mechanism {16b}

Randomization will be performed using a dedicated, password-protected, SSL-encrypted website (Castor EDC, Amsterdam, The Netherlands).

### Implementation {16c}

The allocation sequence is generated by a dedicated computer randomization software program (Castor EDC, Amsterdam, The Netherlands). Patients will be enrolled by local investigators and/or treating physicians in participating ICUs, and the intervention will be randomly assigned by the computer randomization software.

## Assignment of interventions: blinding

### Who will be blinded {17a}

Due to the nature of the intervention, the clinicians and the outcome assessors are not blinded, but the data analysts will remain blinded.

### Procedure for unblinding if needed {17b}

Not applicable, there is no blinding of care providers.

## Data collection and management

### Plans for assessment and collection of outcomes {18a}

Only data needed to assess primary and secondary objectives will be collected in electronic case report forms and extraction from the patient registry systems. Data will be regularly checked on quality, errors, and outliers and corrected if possible.

Two questionnaires are used for the follow-up of subjects from the Netherlands:

- EQ-5D [28, 29]
- A self-developed questionnaire assessing patient opinion and experience of the consent procedure of research in the emergency setting, which is a modified and translated version of the questionnaire used in a previous trial [30].

Subjects will receive these questionnaires per mail or e-mail.

### Plans to promote participant retention and complete follow-up {18b}

No or minimal losses to follow-up for the primary outcome is anticipated. Complete-case analysis will be carried out for all the outcomes. However, if more than 5% of missing data is found for the primary outcome, a sensitivity analysis using multiple imputations will be carried out.

### Data management {19}

All patients will be allocated with a random patient identification code. Patient-identifying data will be omitted. The codebook will be stored digitally and in paper and will be safeguarded by the site investigator. The paper version will be stored behind a lock and the digital form will be encrypted. Source data will be stored at the specific study site where it originated and will be safeguarded by the site investigator. Data sent to the project leader or principal investigator will only contain this code and will not contain patient-identifying information.

### Confidentiality {27}

A codebook of enrolled participants will be collected and stored digitally or in paper, encrypted or behind a lock. The personal information in these files will not be shared with other investigators.

### Plans for collection, laboratory evaluation, and storage of biological specimens for genetic or molecular analysis in this trial/future use {33}

Not applicable, no biological specimens are collected.

## Statistical methods

### Statistical methods for primary and secondary outcomes {20a}

#### Primary outcome

The primary endpoint, all-cause mortality at day 28, is analyzed using Kaplan Meier. The statistical analysis will be based on the intention-to-treat principle, with patients analyzed according to their assigned treatment arms, except for cases withdrawn or without informed consent. The primary outcome will be assessed using a two-sided superiority hypothesis test, with a significance level of 0.05 and presented with two-sided 95% confidence intervals. In addition, we will perform a per-protocol analysis to check for robustness of results. The per-protocol group analysis only considers patients of the conservative group if 50% or more of the PaO_2_s in the blood gas analysis is equal to or below 10.7 kPa (80 mmHg), and patients of the conventional group if 50% or more of the PaO_2_ in the blood gas analysis is equal to or above 14.7 kPa (110 mmHg).

#### Secondary outcome

Secondary endpoints that fall under the category of continuous normally distributed variables will be expressed as frequencies and percentages. Differences between groups in continuous normally distributed variables will be expressed by their means and standard deviations or when not normally distributed, as medians and their interquartile ranges. Secondary endpoints that fall under the category of categorical variables will be expressed as frequencies and percentages. Differences between groups in continuous variables will be analyzed with Student’s *t* test or, if continuous data is not normally distributed, the Mann-Whitney *U* test will be used. Categorical variables will be compared with the chi-squared test or Fisher’s exact test, as appropriate. Statistical significance is considered to be at a *p*-value < 0.05 with a two-sided test. When appropriate, statistical uncertainty will be expressed by 95% confidence levels. In addition to the unadjusted *p*-values for secondary outcomes, a procedure will be applied to control for multiple testing.

All statistical analyses will be performed with the R language and environment for statistical computing (R Foundation for Statistical Computing, Vienna, Austria).

### Interim analyses {21b}

No planned interim analysis will be performed. The data safety monitoring board (DSMB) will analyze a proxy endpoint, in-hospital mortality, for subject safety.

The stopping guidelines are defined as follows: The primary endpoint will be analyzed for safety reasons if a difference in in-hospital mortality of > 6% is found with a *p*-value < 0.005 (chi-square test). The study will only be stopped early for safety reasons if a difference in primary endpoint (28-day mortality) is found of > 6% with a *p*-value of < 0.001.

### Methods for additional analyses (e.g., subgroup analyses) {20b}

Subgroup analyses are planned to investigate the effects of oxygenation targets on the primary endpoint in the following subgroups: ARDS at ICU admission, patients with sepsis as reason for admission, patients with stroke, patients with myocardial infarction, and patients with elevated plasma lactate (> 2 mmol/l).

### Methods in analysis to handle protocol non-adherence and any statistical methods to handle missing data {20c}

Analysis will primarily be performed following the intention-to-treat principle. To handle protocol non-adherence, a secondary per-protocol analysis will be performed.

No or minimal losses to follow-up for the primary outcome is anticipated. Complete-case analysis will be carried out for all the outcomes. However, if more than 5% of missing data is found for the primary outcome, a sensitivity analysis using multiple imputations will be carried out.

### Plans to give access to the full protocol, participant-level data, and statistical code {31c}

The full protocol will be publicly accessible. Upon reasonable request, the dataset and statistical code will be made available.

## Oversight and monitoring

### Composition of the coordinating center and trial steering committee {5d}

The coordinating center and steering committee will provide trial oversight and is composed of the principal investigator, leading investigators, and experts of ventilation who contributed to the design and revision of the study protocol. The leading investigators are responsible for the daily management of the trial and provide assistance to participating ICUs in training in study-related procedures for the local staff, trial management, data management, and monitoring. Local investigators in each site will screen the patients who require mechanical ventilation and check if they are eligible for participation, perform randomization, supervise data collection, and ensure adherence to the ICH-GCP guidelines during the trial.

### Composition of the data monitoring committee, its role and reporting structure {21a}

An independent Data Safety and Monitoring Board (DSMB) watches over the ethics of conducting the study in accordance with the Declaration of Helsinki and monitors safety parameters and the overall conduct of the study. The DSMB is composed of three independent individuals. The DSMB will meet at least yearly. No competing interests were reported by the DSMB.

### Adverse event reporting and harms {22}

Adverse events (AE) are defined as any undesirable experience occurring to a subject during the study, whether or not considered related to the trial procedure and intervention strategies. Since this is a low-risk study in critically ill patients, comparing two currently used PaO_2_ targets, additional undesirable events related to the study protocol are not anticipated. Therefore, we will only register serious adverse events (SAEs) and will not record AEs.

Because this is a study in critically ill patients, SAEs are expected to occur frequently. Therefore, the following SAEs are not considered untoward in this population and will not be treated as SAE:

- Death not related to the study intervention
- Infections
- Bleeding
- Organ failure

The following events occurring during ICU admission will be treated and registered as SAE:

- PaO_2_ ≤ 5 kPa (37.5 mmHg)
- Ischemic events (limbs, cerebral, myocardial, intestinal)
- In-hospital cardiac arrest (IHCA)
- SpO_2_ < 80% for longer than 10 min (not explained by technical failure)
- Death possibly related to the study intervention

The site investigator will report all SAEs to the leading investigator without undue delay after obtaining knowledge of the events.

The sponsor or lead investigator will report the SAEs through the web portal to the accredited ethical reviewing board that approved the protocol, within 7 days of first knowledge for SAEs that result in death or are life threatening followed by a period of maximum of 8 days to complete the initial preliminary report. All other SAEs will be reported within a period of maximum 15 days after the sponsor has first knowledge of the serious adverse events.

### Frequency and plans for auditing trial conduct {23}

On-site monitoring will comprise controlling presence and completeness of the research files and the informed consent forms, source data checks will be performed as described in the monitoring plan. Every participating center will be visited at least once every year.

Monitoring in the Leiden University Medical Center, the coordinating site, will be executed by internal monitors of the LUMC according to the monitor plan. Independent monitoring of participating sites will be arranged by the coordinating investigator and principal investigator.

### Plans for communicating important protocol amendments to relevant parties (e.g., trial participants, ethical committees) {25}

A substantial amendment is defined as an amendment to the terms of the ethical reviewing board application, or to the protocol or any other supporting documentation, that is likely to affect to a significant degree:

- The safety or physical or mental integrity of the subjects of the trial;
- The scientific value of the trial;
- The conduct or management of the trial; or
- The quality or safety of any intervention used in the trial.

All substantial amendments will be notified to the ethical reviewing board and to the competent authority.

### Dissemination plans {31a}

The study protocol and analysis plan will be published before start of the study on trialregister.nl (trial number: 7376). The results of the study will be presented to (inter-) national scientific journals, professional societies, and guideline committees. We will submit analyses to scientific journals in the field of Intensive Care medicine as well as anesthesiology, since both ICU physicians and anesthesiologists apply ventilation in the ICU setting. The results of this study will be disclosed unreservedly according to the Central Committee on Research Involving Human Subjects (CCMO) statement on publication policy (http://www.ccmo.nl/attachments/files/ccmo-statement-publicatiebeleid-3-02-en.pdf). Material for public dissemination will be submitted to the sponsor for review prior to submission for publication. Each study site will provide one co-author, when at least ten subjects have been included. If more than one hundred subjects have been included or reasonable efforts have been made to reach this number the study site will provide two co-authors. The co-authors will be determined in accordance with general accepted academic standards for authorship. Prior to submission, co-authors will look through the manuscript. No parties involved have veto right.

## Discussion

The ICONIC study is a randomized clinical trial that is sufficiently powered to investigate whether a difference in outcome exists between mechanically ventilated ICU patients targeted at conservative or conventional oxygenation. Our aim is to replicate the study that was conducted by Girardis et al., in order to see if we would come to equivocal conclusions. After starting the ICONIC trial, the evidence of the previously mentioned Italian trial [16] and before mentioned studies resulted in clinical practice guidelines that emphasized a more conservative approach of oxygen therapy [6, 22–24, 26]. This encouraged the start of several other randomized trials, including the ICU-ROX, LOCO_2_, the HOT-ICU, and the present trial.

The ICU-ROX investigators compared conservative oxygen therapy (targeting SpO_2_ of 90–96%) to usual care (SpO_2_ > 90%) in 1000 adults undergoing mechanical ventilation in Australia and New Zealand. Conservative oxygen therapy did not improve ventilator-free days or survival in mechanically ventilated adults. However, the interventions compared were conservative oxygen therapy and usual care targeting SpO_2_, and the actual difference in achieved SpO_2_ values between the two groups was minimal. Possibly the chosen target ranges were too close and did not allow sufficient discrimination, reducing the chance to detect any difference in endpoint.

The LOCO_2_ trial planned to randomize 850 French ARDS patients to conservative (target PaO_2_ 55–70 mmHg; target SpO_2_ 88–92%) or liberal oxygen therapy (target PaO_2_ 90–105 mmHg; target SpO_2_ ≥ 96%). However, the trial was stopped prematurely after enrolling 205 patients because of safety concerns due to ischemic events occurring in the conservative group.

Lastly, the most recent published trial from the HOT-ICU group randomized 2928 mechanically ventilated ICU patients to a PaO_2_ of either 60 mmHg or a PaO_2_ of 90 mmHg. No difference in death within 90 days was found. A limitation of this study was that possibly two “normoxia” targets were compared and that there was limited contrast in the applied intervention.

The most recent trials do not support the previously found benefits of conservative oxygen use [16]. Potential explanation for the negative findings in later trials is the lack of contrast between the oxygenation targets (intervention) in both study groups. To add, no truly hyperoxic targets were included in the negative trials. In the literature, hyperoxia or higher targets are either defined as an PaO_2_ of > 100 mmHg, an PaO_2_ > 150 mmHg or even an PaO_2_ of > 300 mmHg [31–35]. In the study by Girardis that did show benefit in the lower oxygenation group, the PaO_2_ target in the control group was up to 150 mmHg, thus more hyperoxic than the oxygenation targets in the negative RCTs.

In order to build on previously published, results we hope to answer questions that remained unanswered in existing literature. Therefore, one of the strengths of the ICONIC is that we chose targets that are further apart, namely 55–80 mmHg vs 110–150 mmHg. To add, to maximize generalizability, we plan to not only focus on ARDS but include patients with a variety of conditions. Due to evidence of ischemia in the conservative group in the LOCO_2_ trial, we will monitor occurrence of ischemic events (cardiac, intestinal, cerebral, and peripheral) closely.

A limitation of this study can be the difficulty for patients to reach their target range. The ability to reach a higher target range highly depends on the lung function and underlying disease. Therefore, it might be possible that a patient is randomized to the higher group but due to underlying condition or clinical deterioration is not able to reach the higher target. We attempted to minimize this risk by excluding patients with ARDS and a P/F ratio < 20, but we can unfortunately not anticipate on the risk of future clinical deterioration. Also patients with healthy lungs that are randomized in the lower oxygenation group might easily reach an SpO_2_ of above 80 mmHg with the slightest additional oxygen. For this reason, patients with an expected duration of ventilation of less than 24 h are also excluded. Another limitation of this study could be that we focus on the whole ICU population instead of subgroups. Suggestions in literature have been made that some subgroups might benefit from a higher or lower oxygenation strategy, but a recent mini-review by Demiselle et al. shows that when pooling the data from different subgroups that still no “optimal” oxygenation target for subgroups can be chosen [36]. Also groups in which a specific oxygen target is proven to be beneficial, for example in COPD patients, were excluded from the study.

In conclusion, the ICONIC study is an investigator-initiated international randomized clinical trial aiming to answer the question how to target oxygen therapy by investigating whether a difference in outcome exists between mechanically ventilated ICU patients targeted at conservative or conventional oxygenation.

## Trial status

Protocol version number: Version 11, 13 February 2020

Date recruitment began: 19 November 2018

Approximate date when recruitment will be completed: 1 January 2022

## Data Availability

Upon reasonable request, the dataset and statistical code will be made available by the principal investigator.

## Abbreviations

AE: Adverse event
ARDS: Acute respiratory distress syndrome
APACHE: Acute Physiology And Chronic Health Evaluation
CCMO: Central Committee on Research Involving Human Subjects; in Dutch: Centrale Commissie Mensgebonden Onderzoek
COPD: Chronic obstructive pulmonary disease
DSMB: Data Safety Monitoring Board
(e)CRF: (Electronic) Case Report Form
FiO_2_: Fraction of inspired oxygen
Hb: Hemoglobin
ICU: Intensive care unit
IHCA: In-hospital cardiac arrest
LOS: Length of stay
LUMC: Leiden University Medical Centre
NICE: National Intensive Care Evaluation
NWO: Dutch research council
PaO_2_: Partial pressure of arterial oxygen
PBW: Predicted body weight
PEEP: Positive end-expiratory pressure
PROVE: PROtective VEntilation
RCT: Randomized clinical trial
SAE: Serious adverse event
SOFA: Sequential Organ Failure Assessment
SpO_2_: Oxyhemoglobin saturation
VA-ECMO: Veno-arterial extracorporeal membrane oxygenation
WMO: Medical Research Involving Human Subjects Act (in Dutch: Wet Medisch-wetenschappelijk Onderzoek met Mensen)
